# Genetic Background May Contribute to PAM50 Gene Expression Breast Cancer Subtype Assignments

**DOI:** 10.1371/journal.pone.0072287

**Published:** 2013-08-28

**Authors:** Ying Hu, Ling Bai, Thomas Geiger, Natalie Goldberger, Renard C. Walker, Jeffery E. Green, Lalage M. Wakefield, Kent W. Hunter

**Affiliations:** 1 Center for Biomedical Informatics and Information Technology, Bethesda, Maryland, United States of America; 2 Laboratory of Cancer Biology and Genetics, CCR, NCI, NIH, Bethesda, Maryland, United States of America; Baylor College of Medicine, United States of America

## Abstract

Recent advances in genome wide transcriptional analysis have provided greater insights into the etiology and heterogeneity of breast cancer. Molecular signatures have been developed that stratify the conventional estrogen receptor positive or negative categories into subtypes that are associated with differing clinical outcomes. It is thought that the expression patterns of the molecular subtypes primarily reflect cell-of-origin or tumor driver mutations. In this study however, using a genetically engineered mouse mammary tumor model we demonstrate that the PAM50 subtype signature of tumors driven by a common oncogenic event can be significantly influenced by the genetic background on which the tumor arises. These results have important implications for interpretation of “snapshot” expression profiles, as well as suggesting that incorporation of genetic background effects may allow investigation into phenotypes not initially anticipated in individual mouse models of cancer.

## Introduction

The past decades have seen significant advances in our understanding of and ability to model breast cancer. The advent of genome-wide expression profiling has led to the development of prognostic gene signatures [1] and improved molecular subtyping [2] that can stratify tumors into groups with different clinical outcomes. These molecular subtypes express signatures similar to those of different cellular components of the mammary duct, including both luminal and basal cell types, and are thought to reflect contributions from the tumor cell type of origin and somatic mutations. The subtyping tools have also been applied to mouse models to gain better understanding of the particular class of breast tumors that the models may represent [3]. Investigators can thus better focus on appropriate models for further characterization or translational studies of breast cancer subtypes of interest. This improved understanding of breast cancer may also permit more sophisticated targeting of particular somatic events associated with the different breast cancer subtypes [4] to the presumptive cell of origin in future mouse models to further improve our understanding of this pervasive disease.

In addition to cell of origin and somatic mutation events, studies over the past 10 years have demonstrated that genetic polymorphism can significantly affect gene expression. Studies in a variety of species have shown that the expression of a significant fraction of the genome can vary across genetically segregating populations [5]. Furthermore genetically-driven variation in gene expression across populations can be observed within tumors with a given driver mutation [6], indicating that inherited as well as somatically acquired changes in gene expression are likely to play an important role in tumor biology. Efforts from our laboratory have been consistent with this interpretation. Inherited variation in individual genes segregating in mouse strains have been associated with multiple tumor phenotypes including tumor latency [7], growth rate [8] and metastatic potential [6], [9]–[11].

The investigations in our laboratory have been based on a single mouse model of metastatic breast cancer, the MMTV-PyMT transgenic model [12]. Originally characterized on the FVB/N mouse strain, this transgenic mouse is considered to be a model of luminal human breast cancer [3], based on hierarchical clustering of the mouse tumors with human breast cancer cell lines, although like most mouse mammary tumors, it does not express ER. The polymorphic genes identified by our earlier work have shown functional relevance only in ER+ human breast cancer samples [13], consistent with the luminal assignment of the MMTV-PyMT model. Unexpectedly, recent results using more genetically diverse mouse strains and novel genetic mapping tools have revealed significant associations that are restricted to estrogen receptor-negative (ER–) human breast cancer. This observation led us to hypothesize that breast cancer subtype assignment based on gene expression patterns in the primary tumor may be partially dependent on genetic background.

To explore this possibility, analysis of the tumor characteristics of genetic crosses between MMTV-PyMT and NZB/B1NJ, a common laboratory strain, MOLF/Ei, a wild-derived mouse strain, and the Diversity Outcross, an outbred population based on eight founder strains [14], was performed. Subtype predictions for the mouse tumors investigated using the PAM50 classifier [15], a gene expression signature capable of classifying breast cancers into classes associated with differing clinical outcomes. Clustering based on the PAM50 subtyping signature demonstrated a distribution of animals across all five human breast cancer subtypes (luminal A, luminal B, HER2, normal-like, basal). Genetic mapping in the mouse populations revealed loci associated with predisposition to PAM50 subtype assignments, consistent with an inherited genetic component. Taken together these results indicate that gene expression based tumor classifiers like the PAM50 capture information beyond simple cell-of-origin and somatic mutational events. Furthermore the results indicate that mouse models may acquire additional characteristics, depending on genetic background, which may extend their utility for modeling human disease.

## Materials and Methods

### Mice

The NZB backcross has been previously described [16]. MOLF backcross animals were generated by breeding MMTV-PyMT male animals to MOLF/EiJ females and subsequently breeding the PyMT-positive F1 males to FVB/NJ females to generate PyMT^+^ N2 females. Diversity Outbred (DO Generation 5, Jackson Laboratory) animals used in this experiment were generated by breeding MMTV-PyMT males to DO females to generate PyMT^+^ F1 females. Animals were housed in groups of 3–5 animals in conventional (MOLF/Ei cross, Fox Chase Cancer Center) or specific pathogen free conditions (DO, NCI) on shaved pine bedding. The animals were maintained on a 12 hour light/dark cycle, with food and water provided *ad libidum*. PyMT^+^ animals were aged to permit tumor development then euthanized as total tumor burden approached the 10% body weight humane endpoint. Individual animals in all of the crosses developed tumors in 6–10 of the mammary glands. Euthanasia was performed by cervical dislocation after Avertin anesthesia. All experiments were performed under protocols approved by the Fox Chase Cancer Center or National Cancer Center Institutional Animal Care and Use Committees. Tumor and metastatic phenotypes were collected as previously described [17]. The incidence of metastases for each of the crosses is indicated in table 1.

**Table 1:** Incidence of Pulmonary Metastases in Mouse Samples.

Cross	Number of animals with metastases	Number of metastasis free animals
DO	72	52
MOLF/Ei	76	90
NZB/B1NJ	51	16

### Gene Expression Analysis

RNA from mammary tumors was isolated using Trizol, following manufacturer’s recommended protocol. The NZB tumors were profiled using Affymetrix MOE430 v2 chips, as described [17] and are available through the Gene Expression Omnibus, accession no. GSE30866. The MOLF and DO tumors were profiled by the NCI Laboratory of Molecular Technology using the Mouse Gene 1.0×ST chip (GSE48566). Data for the Rotterdam breast cancer gene expression samples (GSE2034) were downloaded from the Gene Expression Omnibus (GEO). Expression data for the TCGA samples were downloaded from The Cancer Genome Atlas database (http://cancergenome.nih.gov/).

PAM50 gene listing was from the data sets of the R package genefu [18]. The orthologous genes of PAM50 in the two species, human and mouse were based on the NCBI homology data base. 42 orthologous genes were found to be unambiguously shared in the data sets. The multiple data sets were simply merged into a matrix and the normalization of the merged data sets was performed using R package clusterSim [19]. The range of the normalized values was from −1 to 1.

The subtype prediction of the mouse samples were estimated by the sub-clustering analysis. The hierarchical clustering was performed using the complete linkage algorithm with GSE2034 and one of other 4 data sets. For each hierarchical cluster, the sub-clusters were constructed by the cut heights given the desired number from 2 to 20. The proportions of the GSE2034 subtypes were calculated for each sub-cluster if the number of GSE2034 sample in the sub-cluster is more than 4. The proportion of the GSE2034 subtypes was treated as the probabilities of the mouse and TCGA subtypes. The TCGA genefu prediction was used for algorithm performance analysis.

The normalized data for the PAM50 classifier for all 5 data sets was imported into BRB Array Tools [20]. Visualization of the data was performed using the cluster samples and gene function.

### Immunohistochemistry

Immunohistochemistry staining was performed by the Frederick National Laboratory for Cancer Research Pathology/Histochemistry Laboratory.

## Results

### The PAM50 Intrinsic Subtype Classifier is Significantly Influenced by Genetic Background

Previous studies using network analysis demonstrated that conserved network modules exist between human patient samples and MMTV-PyMT derived mouse tumor samples that are prognostic for distant metastasis and survival [21]. Interestingly, conserved modules were identified that were prognostic for either estrogen receptor positive (ER+) or estrogen receptor negative (ER–) human breast cancer patients. The latter was unexpected since the PyMT mouse mammary tumor model is thought to be a model of a luminal human breast cancer [3]. Since all the mouse tumors were induced by the expression of the same driver event (the PyMT transgene) the different gene expression patterns and modules revealed by the genetic crosses are thought to be significantly influenced by inherited factors that affect gene expression. This therefore suggests that tumor subtype might also be significantly affected by the genetic background on which a tumor arises. In this study we therefore sought to use combined mouse and human resources to determine whether susceptibility to PAM50-defined tumor subtypes might be inherited traits.

**Figure 1:**
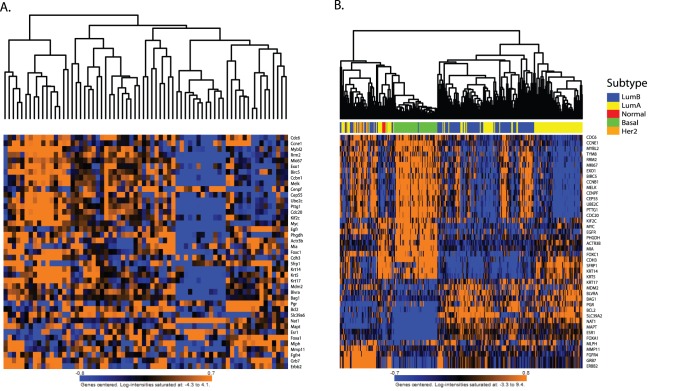
PAM50 clustering. A) NZB cross samples after unsupervised hierarchical clustered using the mouse orthologs of the PAM50 signature. B) The human breast cancer U133A gene expression data set after PAM50 subtype clustering using the Genefu R subtyping algorithm. Subtype classifications are indicated along the top of the heatmap.

To address this hypothesis, unsupervised hierarchical clustering of the gene expression data from an NZB backcross was performed. This backcross expression data set consists of 68 mammary tumors from a genetic mapping backcross performed between NZB/B1NJ and MMTV-PyMT mice [16], arrayed on the MOE430 v2 chip [21]. The data was filtered for the 42 genes comprising the PAM50 intrinsic subtype classifier and clustering performed. As can been observed in figure 1a, distinct subgroups within the mouse samples were observed, reminiscent of the subtypes observed by PAM50 subtyping of the GSE2034 human breast cancer data (figure 1b) [22] set. These results were therefore consistent with the possibility that genetic background significantly contributes to gene expression-based breast cancer subtype assignments.

**Figure 2:**
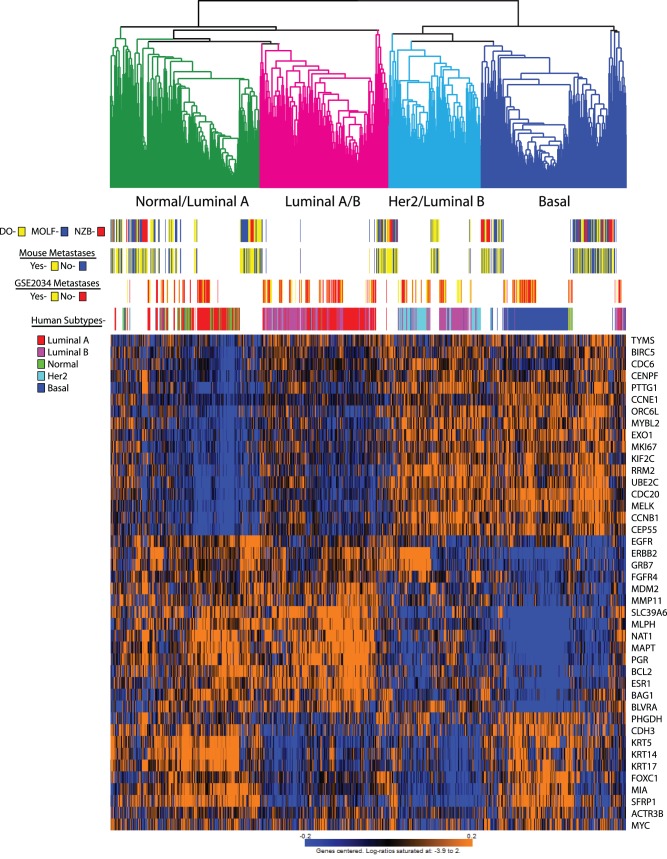
Unsupervised clustering of the GSE2034, TCGA, MOLF, DO and NZB PAM50 genes. The position of the mouse samples and the subtypes of the human samples are indicated across the top of the heatmap. The color coding of the clustering across the top of the figure is based on the enrichment of human samples within each major block.

Further investigation was performed by expanding both the mouse and human data sets. In addition to the small NZB backcross two additional mouse expression data sets were generated. 134 tumors from the previously described MOLF mouse backcross [21] and 133 tumors from a cross between Diversity Outbred (DO) [14], [23] and PyMT were arrayed and included in the analysis. These crosses represent increasing genetic diversity (NZB<MOLF<DO) due to the presence of wild mouse-derived polymorphisms in the MOLF and DO crosses, with the DO cross approximating the degree of polymorphism observed in humans. In addition the 286 human tumor samples from the GSE2034 data set were supplemented by the addition of the TCGA (The Cancer Genome Atlas; N = 466) expression data. Expression data from each of the array platforms (MOE430 v2, Mouse Gene 1.0×ST, U133A, Illumina) were normalized, filtered for the genes comprising the PAM50 subtype signature and unsupervised hierarchical clustering performed. As can be observed in figure 2, mouse tumors from all three data sets distributed across all of the human subtypes, consistent with the possibility that tumor subtype classification by PAM50 signature might be significantly influenced by inherited polymorphism.

### Susceptibility Loci Exist for PAM50 Gene Expression Subtypes

**Figure 3:**
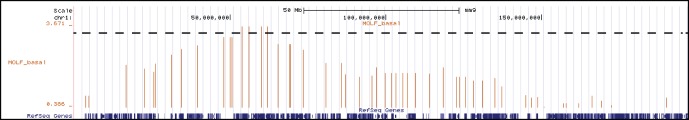
Association mapping of basal tumor susceptibility locus in the MOLF cross. Refseq genes on chromosome 1 are presented along the X-axis. SNPs tested are indicated by the vertical brown lines. Genome wide significance for association with the basal subtype is indicated by the dashed horizontal line.

**Figure 4:**
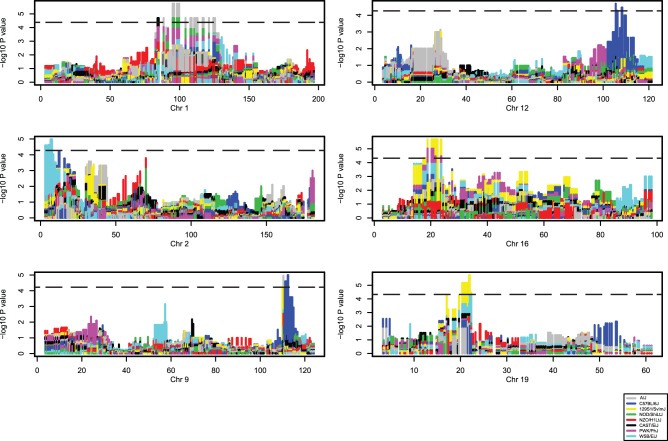
Haplotype associations with the Luminal B/HER2 subtype in the DO cross. Individual chromosomes are depicted on the X-axes. P value for association with tumor subtype is indicated on the Y-axes. Genome wide significance threshold (FDR = 0.05) is indicated by horizontal dashed lines. The strain origin for haplotypes is indicated by color.

The clustering results are consistent with the presence of inherited loci that contribute to subtype gene expression signatures. If true, this suggests that it should be possible to map inherited loci that predispose tumors to assignment to specific intrinsic subtypes. Genotyping of the MOLF and DO mouse populations was therefore performed to attempt to map subtype susceptibility genes. Due to the relatively small number of samples in the NZB cross this data set was not included in this analysis. Genotyping was performed using spleen DNA on either the Illumina Mouse Medium Density Linkage Panel (MOLF, CIDR Genotyping Service) or high density MUGA SNP chip (DO; Genseek Inc.). The genotype data was then screened for SNPs that showed significant associations with subtype assignments based on the unsupervised hierarchical clustering analysis. A single locus on mouse chromosome 1 (53.8–61.9 mb; figure 3) in the MOLF cross was found to be significantly associated with the basal subtype (p = 0.0002, FDR = 0.033). Haplotype-based analysis of the DO cross was performed to identify both regions of the genome associated with subtype assignment and the probably strain of origin of the significant alleles. As can be observed in figure 4 six loci were associated with the HER2/Luminal B assignment in the DO samples as indicated by peaks exceeding the genome-wide significance threshold depicted by the dotted line (Chrs 1, 2, 9, 12, 16, 19; p = 6.39×10^–5^−1.79×10^–6^, FDR = 0.04–0.0073). The different colors of the peaks indicated that multiple DO progenitor strains contributed to the overall HER2/Luminal B susceptibility in this cross.

### Mouse PAM50 Classification is Independent of Conventional Immunohistochemical Markers

**Figure 5:**
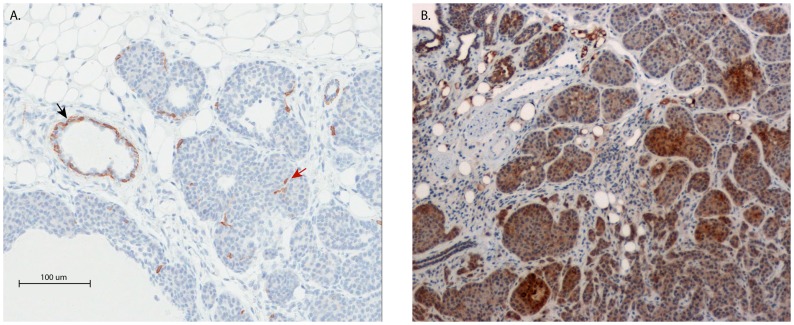
Cytokeratin staining of a representative MOLF basal-like tumors. A) Keratin 5 staining. Positive cells were observed in normal ducts (black arrow) but only occasionally within the tumor mass (red arrow). B) Keratin 8 staining demonstrated heterogeneous staining across tumor samples.

To determine whether the genetic background of the tumors influenced the expression of standard clinical immunohistochemical markers as well as PAM50-defined intrinsic subtypes staining of tumors was performed. Three representative tumors clustering with human basal or luminal A subtypes from the MOLF cross were stained for estrogen or progesterone receptor, Ki67, Her2, the basal cytokeratin marker keratin 5 and the luminal cytokeratin 8. Basal cytokeratin 5 staining was restricted to the basal cells in the normal ducts, with occasional positive cells observed within tumor masses (figure 5A) in all tumors. Heterogeneity of luminal cytokeratin 8 was seen in all tumors, with focal regions of high expression observed in all tumors (figure 5B). Ki67 and Her2 staining was observed throughout all of the samples regardless of subtype prediction. As anticipated from previous studies, no ER or PR staining was observed in any of the tumor samples (data not shown).

### PAM50 Classification is Independent of Metastasis Susceptibility

The PAM50 classifier has been investigated as a tool to improve standard histopathological methods for subdividing breast cancer patients into classes with differing clinical outcomes. Outcome for most epithelial cancers are related to the development of metastatic disease and one might anticipate metastatic tumors would cluster in those subtypes with worst overall outcome. Outcome in human disease however is linked to many different factors, including tumor subtype, age of diagnosis, treatment selection, response to treatment etc. which could obscure potential association between subtype classification and metastatic susceptibility.

To investigate this possibility the association between PAM50 subtypes and distant metastasis free survival was investigated for the GSE2034 human breast cancer data set as well as the mouse samples. The GSE2034 data set consists of lymph node negative breast cancer patients whose tumors were resected but not treated with adjuvant therapy and thus represent the natural history of the disease without systemic therapy. The mouse populations were not subject to any therapeutic intervention and therefore represent the natural course of the disease. As can be observed in figure 2, no clustering of metastasis was observed for either the human or mouse samples (P = 0.56), indicating that the inherited susceptibility for the PAM50-defined tumor subtypes is independent of the inherited susceptibility of developing distant metastatic disease.

## Discussion

Diagnostic and prognostic gene signatures have great potential to improve the ability to stratify patients into appropriate treatment regimens. Improved stratification will enable clinicians to select the most efficacious treatment as first-line therapy which likely will significantly improve patient response and long term outcome. Just as importantly, clinicians would be able to avoid those therapeutic options that would be less effective, sparing patients unnecessary morbidities and toxicities. With these significant advantages it is understandable that a great deal of effort has been spent in developing and validating molecular signatures for clinical use (exs [24]–[26]).

One issue regarding these signatures however is their significance in regard to molecular and cellular origins of the transcriptional profiles. In general, these gene expression profiles are generated by supervised analysis of one set of patient samples, followed by validation in an independent set of samples. The signatures are therefore based on correlations with clinical data or outcomes rather than on molecular events. Any mechanistic basis ascribed to the generation of the signatures is therefore usually by correlation of the expression patterns to known expression patterns that provide plausible explanations.

The lack of experimental validation of the molecular basis of these signatures does not in any way detract from their potential clinical value. However, comprehensive understanding of what induces these transcriptional profiles might reveal important biological insights into disease biology. In this study we have explored some of the factors that may contribute to one of these clinically relevant signatures, the PAM50 breast cancer subtype profiling tool [15]. The conventional interpretation of this signature has been that subtype specific gene signatures are produced in tumors based on the cell of origin [2] and subtype specific mutational events [4]. Unlike human tumors, the mouse tumors generated in our mouse populations all result from the expression of polyoma middle T antigen, driven from the mouse mammary tumor virus enhancer and promoter. In addition, comparative genome hybridization [27] and spectral karyotyping [28] indicate that the genome of mouse PyMT tumors are much more stable than human tumors, likely reducing any contribution of somatic mutation to the observed expression profiles. Furthermore, while we cannot definitively rule out the possibility that varying the genetic background of the host might alter the transcriptional patterns of the transgene, there is no evidence at present to support this possibility.

Thus, if these results can be extrapolated into human patients, subtype assignments based on gene expression alone may reflect a combination of cell of origin, somatic genetic alteration spectrum, and an inherited predisposition to develop tumors with a particular subtype signature. The data from this study suggests that inherited polymorphism may be an additional contributing factor to the establishment of the PAM50 signatures. Moreover, the data indicate that pre-disposition for particular classes of breast cancer likely exist in human populations. Intriguingly, recent studies in human epidemiology support this interpretation, with the demonstration of the existence of loci pre-disposing women to triple negative breast cancer, a subset of basal tumors [29]. Thus by identifying and characterizing the inherited factors that contribute to these signatures, additional unrealized potential of the PAM50 and other clinical profiling tools may be uncovered for understanding the etiology of breast cancer.

Finally, this study also has important implications for the use of animal models for cancer research. Current strategies are to generate individual models based on single or combinations of mutations with the aim to model a particular breast cancer subtype. Each of these models is generally explored on a single genetic background. The results from this study indicate that varying the genetic background gives the ability to investigate additional biological questions for a particular genetically engineered model. This is most clearly evidenced by our ability to identify susceptibility loci associated with ER– breast cancers using a model thought to most closely represent an ER+ luminal subtype. By incorporating genetic background as a variable in research strategies individual mouse models of cancer may therefore have additional utility to explore the complex biology than originally appreciated.

## References

[1] van’t Veer LJ, Dai H, van de Vijver MJ, He YD, Hart AA, et al (2002) Gene expression profiling predicts clinical outcome of breast cancer. Nature 415: 530–536. 11823860 10.1038/415530a

[2] Perou CM, Sorlie T, Eisen MB, van de Rijn M, Jeffrey SS, et al (2000) Molecular portraits of human breast tumours. Nature 406: 747–752. 10963602 10.1038/35021093

[3] Herschkowitz JI, Simin K, Weigman VJ, Mikaelian I, Usary J, et al (2007) Identification of conserved gene expression features between murine mammary carcinoma models and human breast tumors. Genome Biol 8: R76. 17493263 10.1186/gb-2007-8-5-r76 PMC1929138

[4] Cancer Genome Atlas Network (2012) Comprehensive molecular portraits of human breast tumours. Nature 490: 61–70. 23000897 10.1038/nature11412 PMC3465532

[5] Schadt EE, Monks SA, Drake TA, Lusis AJ, Che N, et al (2003) Genetics of gene expression surveyed in maize, mouse and man. Nature 422: 297–302. 12646919 10.1038/nature01434

[6] Crawford NP, Walker RC, Lukes L (2008) Officewala JS, Williams RW, et al (2008) The Diasporin Pathway: a tumor progression-related transcriptional network that predicts breast cancer survival. Clin Exp Metastasis 25: 357–369. 18301994 10.1007/s10585-008-9146-6 PMC2410042

[7] Le Voyer T, Lu Z, Babb J, Lifsted T, Williams M, et al (2000) An epistatic interaction controls the latency of a transgene-induced mammary tumor. Mamm Genome 11: 883–889. 11003704 10.1007/s003350010163

[8] Le Voyer T, Rouse J, Lu Z, Lifsted T, Williams M, et al (2001) Three loci modify growth of a transgene-induced mammary tumor: suppression of proliferation associated with decreased microvessel density. Genomics 74: 253–261. 11414753 10.1006/geno.2001.6562

[9] Park YG, Zhao X, Lesueur F, Lowy DR, Lancaster M, et al (2005) Sipa1 is a candidate for underlying the metastasis efficiency modifier locus Mtes1. Nat Genet 37: 1055–1062. 16142231 10.1038/ng1635 PMC2140048

[10] Crawford NP, Alsarraj J, Lukes L, Walker RC (2008) Officewala JS, et al (2008) Bromodomain 4 activation predicts breast cancer survival. Proc Natl Acad Sci U S A 105: 6380–6385. 18427120 10.1073/pnas.0710331105 PMC2359777

[11] Crawford NP, Qian X, Ziogas A, Papageorge AG, Boersma BJ, et al (2007) Rrp1b, a new candidate susceptibility gene for breast cancer progression and metastasis. PLoS Genet 3: e214. 18081427 10.1371/journal.pgen.0030214 PMC2098807

[12] Guy CT, Cardiff RD, Muller WJ (1992) Induction of mammary tumors by expression of polyomavirus middle T oncogene: A transgenic mouse model for metastatic disease. MCB 12: 954–961. 1312220 10.1128/mcb.12.3.954-961.1992 PMC369527

[13] Hsieh SM, Look MP, Sieuwerts AM, Foekens JA, Hunter KW (2009) Distinct inherited metastasis susceptibility exists for different breast cancer subtypes: a prognosis study. Breast Cancer Res 11: R75. 19825179 10.1186/bcr2412 PMC2790856

[14] Svenson KL, Gatti DM, Valdar W, Welsh CE, Cheng R, et al (2012) High-resolution genetic mapping using the Mouse Diversity outbred population. Genetics 190: 437–447. 22345611 10.1534/genetics.111.132597 PMC3276626

[15] Parker JS, Mullins M, Cheang MC, Leung S, Voduc D, et al (2009) Supervised risk predictor of breast cancer based on intrinsic subtypes. J Clin Oncol 27: 1160–1167. 19204204 10.1200/JCO.2008.18.1370 PMC2667820

[16] Hunter KW, Broman KW, Voyer TL, Lukes L, Cozma D, et al (2001) Predisposition to efficient mammary tumor metastatic progression is linked to the breast cancer metastasis suppressor gene Brms1. Cancer Res 61: 8866–8872. 11751410

[17] Faraji F, Pang Y, Walker RC, Nieves Borges R, Yang L, et al (2012) Cadm1 is a metastasis susceptibility gene that suppresses metastasis by modifying tumor interaction with the cell-mediated immunity. PLoS Genet 8: e1002926. 23028344 10.1371/journal.pgen.1002926 PMC3447942

[18] Walesiak M, Dudek A (2012) Searching for optimal clustering procedure for a data set.

[19] Haibe-Kains B, Schroeder M, Bontempi G, Sotiriou C, Quakenbush J (2012) genefu: Relevant Functions for Gene Expression Analysis, Especially in Breast Cancer.

[20] Simon R, Radmacher MD, Dobbin K, McShane LM (2003) Pitfalls in the use of DNA microarray data for diagnostic and prognostic classification. J Natl Cancer Inst 95: 14–18. 12509396 10.1093/jnci/95.1.14

[21] Hu Y, Wu G, Rusch M, Lukes L, Buetow KH, et al (2012) Integrated cross-species transcriptional network analysis of metastatic susceptibility. Proc Natl Acad Sci U S A 109: 3184–3189. 22308418 10.1073/pnas.1117872109 PMC3286991

[22] Wang Y, Klijn JG, Zhang Y, Sieuwerts AM, Look MP, et al (2005) Gene-expression profiles to predict distant metastasis of lymph-node-negative primary breast cancer. Lancet 365: 671–679. 15721472 10.1016/S0140-6736(05)17947-1

[23] Churchill GA, Gatti DM, Munger SC, Svenson KL (2012) The diversity outbred mouse population. Mamm Genome 23: 713–718. 22892839 10.1007/s00335-012-9414-2 PMC3524832

[24] Kunz G (2011) Use of a genomic test (MammaPrint) in daily clinical practice to assist in risk stratification of young breast cancer patients. Arch Gynecol Obstet 283: 597–602. 20383789 10.1007/s00404-010-1454-9

[25] Zbytek B, Cohen C, Wang J, Page A, Williams DJ, et al (2013) Nottingham-defined mitotic score: comparison with visual and image cytometric phosphohistone H3 labeling indices and correlation with Oncotype DX recurrence score. Appl Immunohistochem Mol Morphol 21: 48–53. 22495373 10.1097/PAI.0b013e3182427cda

[26] Martin M, Prat A, Rodriguez-Lescure A, Caballero R, Ebbert MT, et al (2013) PAM50 proliferation score as a predictor of weekly paclitaxel benefit in breast cancer. Breast Cancer Res Treat 138: 457–466. 23423445 10.1007/s10549-013-2416-2 PMC3608881

[27] Borowsky AD, Namba R, Young LJ, Hunter KW, Hodgson JG, et al (2005) Syngeneic mouse mammary carcinoma cell lines: two closely related cell lines with divergent metastatic behavior. Clin Exp Metastasis 22: 47–59. 16132578 10.1007/s10585-005-2908-5

[28] Montagna C, Lyu MS, Hunter K, Lukes L, Lowther W, et al (2003) The Septin 9 (MSF) gene is amplified and overexpressed in mouse mammary gland adenocarcinomas and human breast cancer cell lines. Cancer Res 63: 2179–2187. 12727837

[29] Stevens KN, Vachon CM, Couch FJ (2013) Genetic susceptibility to triple-negative breast cancer. Cancer Res 73: 2025–2030. 23536562 10.1158/0008-5472.CAN-12-1699 PMC3654815

